# Connexin 37 Regulates the Kv1.3 Pathway and Promotes the Development of Atherosclerosis

**DOI:** 10.1155/2022/2689918

**Published:** 2022-09-23

**Authors:** Minqi Liao, Lihua Chen, Jiongbin Lu, Guangzhu Liang, Yongzhao Yao, Shumin Ouyang, Yanhua Yang, Zhengwei Jian, Suxia Guo

**Affiliations:** Department of Cardiology, Affiliated Dongguan Hospital, Southern Medical University, 523000, China

## Abstract

**Objective:**

To investigate the mechanism of Connexin 37 (Cx37) and Kv1.3 pathways in atherosclerosis (AS).

**Methods:**

ApoE^−/−^ mice were given a high-fat diet to establish atherosclerosis (AS) model, and macrophages in mice were isolated and extracted to transfect Cx37 vectors with silencing or overexpressing, and Kv1.3 pathway blockers were used to inhibit the pathway activity. The indexes of body weight, blood glucose, and blood lipid of mice were collected. The protein and mRNA expression levels of Cx37 and Kv1.3 were detected by reverse transcription-PCR (RT-PCR), Western blot, and immunofluorescence technique. Oil red O staining was used to observe plaque area. Masson staining was used to detect collagen content. The concentrations of chemokine CCL7 were quantified using the ELISA kits. CCK8 was used to detect cell proliferation.

**Results:**

Cx37 and Kv1.3 were highly expressed in macrophages of AS mice, and the expression of Kv1.3 and CCL7 decreased after Cx37 was silenced, and the proliferation of macrophages was also decreased. Wild-type mice and AS model mice were treated with Cx37 overexpression vectors and Kv1.3 pathway blocking, and it was found that Cx37 overexpression could improve the blood lipid and blood glucose levels and increase the area of AS in AS mice. However, blocking the activity of Kv1.3 pathway can reduce the levels of blood lipid and blood glucose, increase the body weight of mice, and reduce the area of AS mice. Blocking the activity of Kv1.3 pathway can slow down the plaque development of AS mice and make its indexes close to wild-type mice. And the use of Kv1.3 pathway blockers on the basis of overexpression of Cx37 indicated that inhibition of Kv1.3 pathway activity did not affect the expression of Cx37, but could inhibit the collagen content in the plaque area of AS mice, inhibit the expression of chemokine CCL7, and reverse the effect of Cx37 overexpression.

**Conclusion:**

Cx37 can improve the activity of macrophages by regulating the expression of chemokines and the activity of Kv1.3 pathway in AS mice, and enrich macrophages in inflammatory tissues and expand the area of plaque formation.

## 1. Background

Atherosclerosis (AS) is a chronic inflammatory disease characterized by immune activity, which is prone to plaque rupture and thrombosis, causing a series of cardiovascular events and seriously harming human health [1]. Anticoagulant, antiplatelet, control of blood pressure, blood lipid and blood sugar are the main methods to prevent and treat atherosclerosis. [2]. The use of the above-mentioned treatments can significantly reduce the occurrence of cardiovascular events, but atherosclerosis is still the primary cause of coronary heart disease and stroke, it accounting for half of the death causes in western countries [3]. It is still a great challenge for cardiovascular therapy to clarify its mechanism and seek more effective treatment measures.

The unstable development of atherosclerotic plaque forms vulnerable plaque, and then, the rupture of vulnerable plaque leads to platelet aggregation and thrombosis, and the infiltration of a large number of inflammatory cells inside plaque is the main cause of plaque rupture, resulting in myocardial ischemia and infarction [4, 5]. With the further study of vulnerable plaques, we found that mononuclear/macrophages are one of the main inflammatory cells in plaque, which not only exist inside of the plaque but also infiltrate the vascular bed outside the artery in large numbers [6]. Under the stimulation of ox LDL, monocyte-derived macrophages are activated to aggregate, adhere, and migrate to vascular walls through endothelial cells and secrete cytokines, chemokines, metalloproteinases, and other proteolytic enzymes to accelerate the activation of inflammatory responses. At the same time, as the inflammatory response worsens, macrophages continue to activate and trigger further inflammatory response, which amplifies the inflammatory response [7]. Therefore, macrophage activation plays an important role in the pathogenesis of emergency of geriatric cardiology.

Connexin 37 (Cx37) is an important protein that mediates inflammatory response which is mainly expressed in vascular endothelial cells. Cx37 expression can also be detected on the surface of foam cells and smooth muscle cells in atherosclerotic plaques [8]. Recent studies have shown that Cx37 is also expressed on the surface of free mononuclear/macrophages in blood. Previous studies have found that Cx37 gene expression is strongly correlated with the incidence of coronary heart disease [9]. In order to further clarify the role of Cx37 in AS, the researchers selected miniature pigs with AS plaque properties highly similar to those of humans for in vivo experiments and found that interference of Cx37 gene expression with siRNA Cx37 transfection resulted in the reduction of AS plaque volume and thickening of fibrous cap, reversing the formation of unstable plaques [10]. These results suggest that Cx37 may play an important role in the development of AS vulnerable plaques, and inhibition of its expression can reverse vulnerable plaques. In the follow-up study, the researcher further observed the effect of siRNA Cx37 transfection on the fractional fow reserve (FFR) of porcine myocardium, and the results showed that after Cx37 siRNA interference, FFR was significantly improved [11], suggesting that inhibition of Cx37 expression can inhibit the progression of AS and improve overall cardiac function. Therefore, the correlation between Cx37 and AS has been confirmed from clinical disease genetics detection and animal experiments, and Cx37 is expected to become a potential target to curb the occurrence and development of AS.

In recent years, great progress has been made in the study of potassium ion channels in macrophages, and it has been found that the potassium ion channels on the surface of macrophages have an extremely important relationship with cell activation. The opening of voltage-dependent potassium channels causes hyperactivation of the cell membrane, promotes Ca^2+^ influx, and indirectly regulates cell proliferation and cytokine secretion [12]. Meanwhile, Kv channel plays a key role in regulating cell volume, electrolyte transport, cell excitation and contraction, enzyme activation, and other life activities [13]. After the activation of macrophages, the potassium channel kv1.3 on the membrane of macrophages affects the activation and function of macrophages by regulating the membrane potential pathway. Activation of macrophages runs throughout atherosclerosis [14–16]. Therefore, there are many studies on affect the activity of macrophages by blocking the Kvl.3 potassium channel.

Therefore, based on the results of previous studies, we hypothesized that Cx37 may regulate the development of AS by regulating the kv1.3 channel. In this study, ApoE^−/−^ mice were used to establish AS model, change the expression of Cx37, and explore the role of Cx37 and KVL.3 channels in AS mice.

## 2. Materials and Methods

### 2.1. Construction of Cx37 Expression Vector

The target DNA fragments were collected, and the Cx37 silenced expression vector (shRNA-1/2/3) was constructed using pLVX-shRNA2-PURO vector plasmid (Hunan Fenghui Biotechnology Co., Ltd.). Meanwhile, the Cx37 overexpression vector (pre-CX37) was constructed using rAAV2/8 vector (Brain Case Biotechnology Co., Ltd., Shenzhen). The silenced expression vector was transfected into 293T cells, and the stably transfected cell lines were constructed by subculture. Subsequently, the protein was extracted, and Western blot was performed to detect the expression efficiency of Cx37. The sequence of Cx37 silenced expression vector is as follows: mCx37-1 sequence: 5′-GCCATCCAAGGACCTACATGT-3′; mCx37-2 sequence: 5′-GCTCATGGGTACCTATGTGGT-3′; mCx37-3 sequence: 5′-GCTCTCATCCACTGAGCAGAA-3′.

### 2.2. Experimental Animal

Eight-week-old ApoE^−/−^ mice were purchased from Beijing Weitong Lihua Company, and C57BL/6 wild-type mice were purchased from Experimental Animal Center of Southern Medical University. All laboratory animals were approved by the Laboratory Animal Ethics Committee of Dongguan People's Hospital and in accordance with the guidelines of the National Institutes of Health for the care and use of laboratory animals.

**Figure 1:**
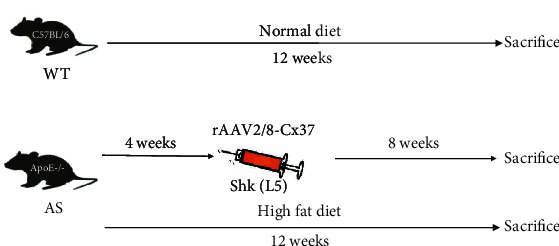
Experimental mouse operation flowchart.

Before the experiment, all animals were fed standard diet with ambient temperature of 20-25°C, humidity of 30-70%, and light/dark for 12 h each. Before administration, all C57BL/6 mice were given a normal diet for 4 weeks, and all ApoE^−/−^ mice were given a high-fat diet for 4 weeks to establish AS model. From week 4, C57BL/6 wild-type mice were fed a normal diet and given an intraperitoneal injection of equal volume of normal saline. ApoE^−/−^ control mice were given high-fat diet and given an intraperitoneal injection of equal volume of normal saline. ApoE^−/−^ mice in pre-CX37 group were given high-fat diet and tail vein injection of Cx37 overexpression vector (pre-CX37) solution. ApoE^−/−^ mice in ShK(L5) group were given high-fat diet and subcutaneous injection of Kv1.3 blocker ShK(L5). During the feeding period, the changes of body weight, blood lipid, and blood glucose of mice in each group were recorded. After 8 weeks of drug feeding, mice in each group were anesthetized and sacrificed, and blood samples and aorta samples of mice were quickly separated and preserved. Experimental operation is shown in Figure 1.

### 2.3. Handling of Experimental Specimens

The mice were sacrificed after blood collection, and aorta was immediately separated. Vascular rings about 0.5 cm long were cut from thoracic aorta, fixed with 4% paraformaldehyde, embedded with paraffin, and finally sected. 50~100 mg of other aortic tissues were selected and frozen in liquid nitrogen. Mouse arterial blood was collected, and monocytes were isolated from mouse peripheral blood by density gradient centrifugation and wall adhesion method [17]. The isolated monocytes were cultured in complete medium and differentiated into macrophages after 5 days of culture.

### 2.4. Western Blot

Cells were collected, and total protein was extracted. BCA solution (FUDE Biology, Hangzhou) was prepared to detect protein concentration of the samples to be tested. The prepared protein samples were taken out, dissolved on the ice box, and then, added into the electrophoresis tank for 40 min. The PVDF membrane of corresponding size was cut, and the separation glue was closely attached to the PVDF membrane, and the protein was transferred to the PVDF membrane in Bio-Rad Trans-Blot. After membrane transfer, remove PVDF membrane, wash it for 3 times, and add an appropriate amount of 5% skim milk powder for 1 h. The membrane was washed with TBS-T for 3 times, and the corresponding strip was added into the primary antibody solution and incubated overnight at 4°C. The next day, the strips were washed with TBS-T for 3 times and then added with corresponding secondary antibodies and incubated at 4°C for 4 h. ECL luminescent solution (Omiget Pharmaceutical Technology Co., Ltd., Beijing) was prepared and uniformly dropped on PVDF membrane, and the reaction was carried out in the darkroom for several seconds. The cut film is completely covered on PVDF membrane, exposed for 5-10 seconds, then put into the prepared developer, using IPP6.0 software for gray value analysis of the film.

### 2.5. Reverse Transcription-PCR

Total RNA of macrophages was extracted with TRIzol reagent (Tiangen Company, Beijing), and cDNA was synthesized by reverse transcription using PrimeScript RT Reagent Kit (TAKARA Company). RT-PCR was then performed using SYBR Premix Ex Taq Kit (TAKARA Company). The primers used are shown in Table 1.

### 2.6. Oil Red O Staining

First, 0.5% oil red O stock solution (Sigma) was prepared, diluted with distilled water (dilution ratio 3 : 2), and stood at room temperature for 10 min. The blood vessel tissue were fixed in 10% neutral formaldehyde solution for 30 min, then removed and bathed in water for 15 min. Tissue samples were added to oil red O solution and stained for 1~2 h. The tissue samples were rinsed with 70% ethanol until the patches were red and the background was white. Finally, the specimen was washed with distilled water, and the staining was observed using a microscope (Shanghai Caikon Optical Instrument Co., Ltd.).

### 2.7. Masson Staining

Paraffin sections of aortic tissue were taken for dewaxing and hydration. Then, the slices were stained with hematoxylin solution for 5-10 min. The sections were then rinsed with running water and stained in ponceau acid fuchsin for 5-10 min. Sections were rinsed again and treated with 1% phosphomolybdic acid aqueous solution for 5 minutes, followed by aniline blue for 5 minutes. Finally, the tissue sections were treated with 1% glacial acetic acid for 1 minute, dehydrated repeatedly with 95% alcohol, and sealed with xylene and neutral gum. The staining was observed using a microscope.

### 2.8. Enzyme-Linked Immunosorbent Assay (ELISA)

Mouse MCP3 ELISA Kit (CCL7) (Abcam) was used to perform the experiment according to the instructions. CCL7 antibody was diluted with carbonate buffer and added to the culture plate for overnight culture at 4°C. The next day, the solution in the culture wells was discarded and washed with PBS solution three times, and the cell samples to be tested were added to the culture wells. The cells were incubated at 37°C for 1 hour. Biotinylated antibody working solution and enzyme conjugate were added to the reaction well, and the reaction well was cleaned after incubation for 30 min. Add color substrate TMB 100 *μ*L to each well, and incubate in a dark room for 15 min. Add stop solution 100 *μ*L to each well, measure OD_450_ value after mixing, and draw standard curve to calculate the concentration.

### 2.9. CCK8

Logarithmic growth stage cells were collected and counted, cell density was adjusted, and cells were inoculated on 96-well plates and incubated overnight at 37°C. 0.1 mL complete culture medium containing 10% CCK8 (Beyotime Institute of Biotechnology, Beijing) was added to each well, and incubated at 37°C and 5% CO_2_ for 2-3 h. OD450 was determined using a microplate reader (Beijing Pulang New Technology Co., Ltd.).

### 2.10. Immunofluorescence Technique

The blood vessel rings to be measured were fixed with methanol for 20 min, and the tissues were dried at room temperature for 10 min. The vascular tissues were cleaned 3 times with PBS solution and treated with 1% Triton solution for 25-30 min. The tissue was then placed in goat serum and sealed at 37°C for 20 min. The tissues were then placed into primary antibody solutions such as Cx37 antibody and Kv1.3 antibody (Abcam) and incubated at 4°C overnight. Then, the tissue was added into a fluorescence-labeled secondary antibody solution (Abcam) for 1 h reaction. After washing the tissue with PBS solution for 3 times, the slides were dried and sealed, and the staining results were observed by fluorescence microscope.

### 2.11. Statistical Analysis

SPSS 20.0 software was used for data analysis. All experiments were set up with three double wells and repeated at least three times. Measurement data were expressed as mean ± standard deviation (‾*x* ± *s*). Continuous variables between the two groups were analyzed by independent sample *t* test, and the mean values between multiple groups were compared by ANOVA analysis. *P* ≤ 0.05 was considered statistically significant.

## 3. Results

### 3.1. Expression of Cx37 and Kv1.3 in Atherosclerosis

**Figure 2:**
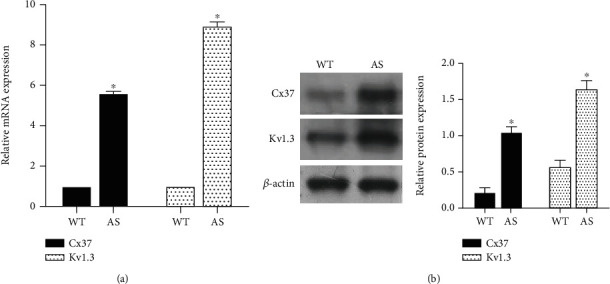
Expression of Cx37 and Kv1.3 in atherosclerosis. (a) The expression of Cx37 and Kv1.3 in macrophages was detected by RT-PCR. (b) The expressions of Cx37 and Kv1.3 proteins were detected by Western blot. ^∗^*P* < 0.05, compared with WT group.

ApoE^−/−^ mice were given a high-fat diet to establish AS mouse model, and macrophages were isolated from WT mice and AS mice to detect the expressions of Cx37 and Kv1.3. The detection results showed that Cx37 and Kv1.3 were highly expressed in macrophages of AS mice (Figure 2(a)). Protein detection also showed that Cx37 and Kv1.3 proteins were highly expressed in AS mice (Figure 2(b)).

### 3.2. Effect of Cx37 Gene on Proliferation and Chemotactic Function of Mononuclear Macrophages

**Figure 3:**
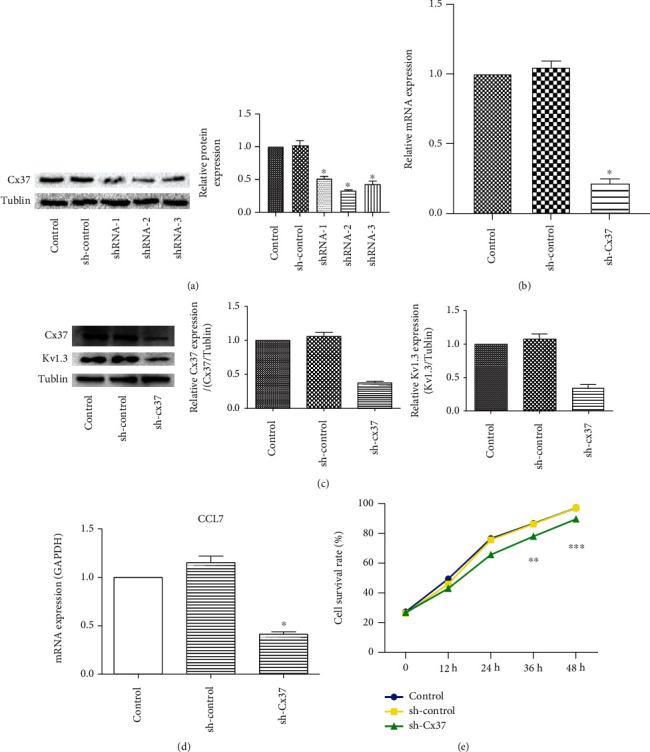
Effect of Cx37 silencing expression on physiological function of mononuclear macrophages. (a) Western blot was used to detect the expression efficiency of Cx37 silenced expression vector. (b) The expression efficiency of Cx37 in macrophages was detected by RT-PCR. (c) The expressions of Cx37 and Kv1.3 proteins were detected by Western blot. (d) The expression of CCL7 was detected by RT-PCR. (e) CCK8 was used to detect cell proliferation. ^∗^*P* < 0.05, ^∗∗^*P* < 0.01, ^∗∗∗^*P* < 0.001, compared with control group.

It has been known from previous studies that Cx37 is highly expressed in AS mononuclear macrophages, and the expression of Cx37 is closely related to the development of AS. Therefore, we silenced the expression of Cx37 in macrophages, constructed Cx37 silenced expression vector, and detected its expression efficiency by WB. It was found that all three expression vectors could effectively inhibit the expression of Cx37, and shRNA-2 had the highest inhibition efficiency (Figure 3(a)). Therefore, we transfected macrophages with shRNA-2 silencing expression vector. RT-PCR results showed that Cx37 expression decreased in sh-CX37 group, and transfection was successful (Figure 3(b)). Subsequently, we detected that reduced Cx37 expression in macrophages resulted in decreased Kv1.3 protein expression and decreased chemokine CCL7 concentration (Figures 3(c) and 3(d)). Meanwhile, silencing Cx37 expression can also inhibit the proliferation activity of macrophages (Figure 3(e)). The expression of Cx37 can promote macrophage activity.

### 3.3. Effect of Cx37 Gene and Kv1.3 Channel on Vein Atherosclerosis in Mice

**Figure 4:**
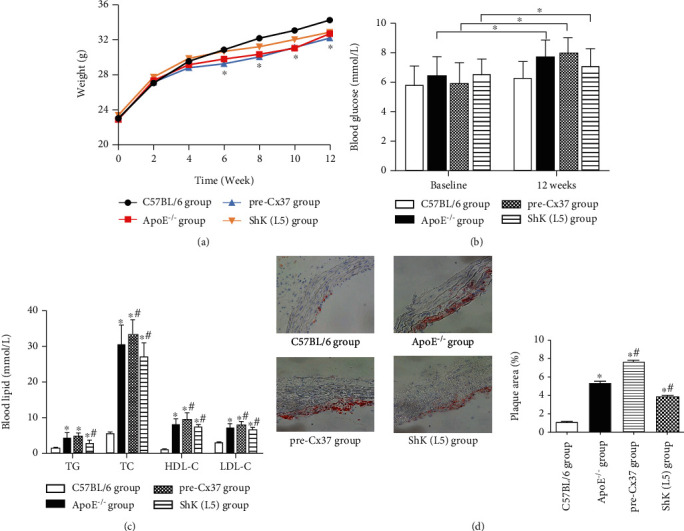
Effect of Cx37 and Kv1.3 expression on atherosclerosis. (a) Body weight statistics of mice. (b) Blood glucose index statistics of mice. (c) Four statistics of blood lipid in mice. (d) The atherosclerotic plaque area was detected by oil red O staining. ^∗^*P* < 0.05, compared with C57BL/6 group; ^#^*P* < 0.05, compared with ApoE^−/−^ group.

ApoE^−/−^ mice were given a high-fat diet, followed by a tail vein injection of Cx37 overexpression vector (pre-CX37) or subcutaneous injection of Kv1.3 blocker ShK (L5). The weight of mice and other indicators were detected, and it was found that there was no significant difference in the weight of mice in the 4 groups in the first 4 weeks. From the 4th week of administration, the rate of weight gain of all AS mice decreased, and the body weight of mice was smaller than that of wild-type mice. There was no significant difference in the weight growth trend between the pre-CX37 group and the ApoE^−/−^ group, and the weight gain of the ShK (L5) group was slightly higher than that of the ApoE^−/−^ group, which was closer to that of the C57BL/6 group (Figure 4(a)).

After 12 weeks, the blood glucose indexes of ApoE^−/−^ mice in three groups were all higher than before. After 12 weeks, the blood glucose index of mice in the pre-CX37 group was higher than that in the ApoE^−/−^ group, and the blood glucose concentration of mice in the ShK(L5) group was lower and close to that of wild-type mice (Figure 4(b)). The four tests of blood lipid in mice showed that the concentrations of TG (triglyceride), TC (total cholesterol), HDL-C (high-density lipoprotein cholesterol), and LDL-C (low-density lipoprotein cholesterol) of AS mice in the three groups were significantly higher than those of wild-type mice, while the four indexes of ShK(L5) group were lower than those of ApoE^−/−^ group and pre-CX37 (Figure 4(c)). Cx37 overexpression has no significant effect on body weight of AS mice, but blocking the activity of Kv1.3 pathway in mice can effectively reduce the increase of blood lipid and blood glucose of AS mice. Oil red O staining results showed that Cx37 overexpression increased the atherosclerotic plaque area, while ShK(L5) decreased the atherosclerotic plaque area (Figure 4(d)). These results suggest that Cx37 overexpression in AS mice can promote the formation of AS plaques, while blocking the activity of Kv1.3 pathway can inhibit the formation of AS plaques.

### 3.4. Study on the Mechanism of Cx37 Gene Promoting the Development of Atherosclerosis by Promoting Kv1.3 Opening

**Figure 5:**
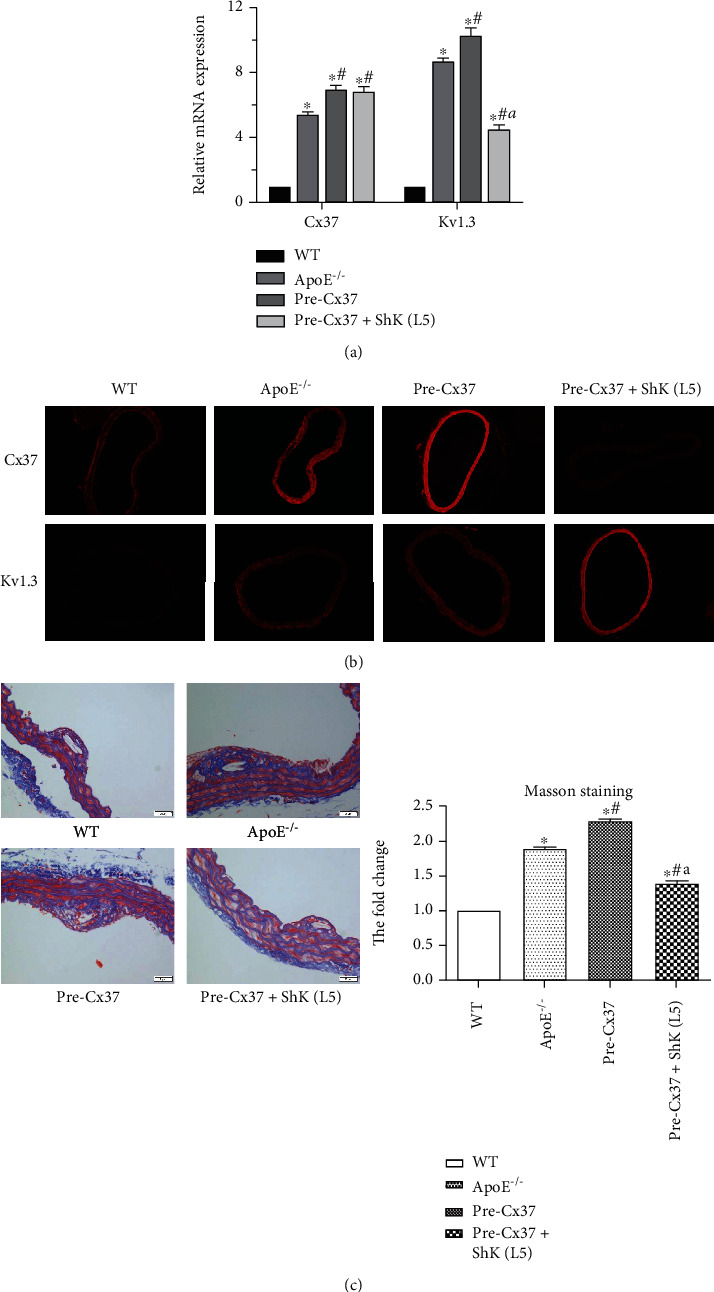
Study on the mechanism of Cx37 gene regulating atherosclerosis. (a) The expression efficiency of Cx37 and Kv1.3 was detected by RT-PCR. (b) The expression of Cx37 and Kv1.3 in mouse aorta was detected by immunofluorescence. (c) The expression of plaque collagen content was detected by Masson staining. ^∗^*P* < 0.05, compared with WT group; ^#^*P* < 0.05, compared with ApoE^−/−^ group; ^a^*P* < 0.05, compared with Pre-Cx37 group.

In order to detect the relationship between Cx37 and Kv1.3 pathway, we selected to add Kv1.3 blocker to culture mice based on Cx37 overexpression. Firstly, we detected that both Kv1.3 and Cx37 were highly expressed in AS mice. After the expression of Cx37 was increased, the expression of Kv1.3 was also increased; however, when the expression of Kv1.3 was inhibited, the expression of Cx37 was not significantly changed (Figure 5(a)). Immunofluorescence detection also showed the same results as RT-PCR: Cx37 overexpression could increase Kv1.3 expression, but decreased Kv1.3 expression could not affect Cx37 expression (Figure 5(b)). Masson staining showed that plaque collagen content in the ApoE^−/−^ group was increased compared with that in the wild-type group. Compared with ApoE^−/−^ group, Cx37 overexpression increased plaque collagen content, and the addition of Kv1.3 blocker on the basis of Cx37 overexpression decreased plaque collagen content (Figure 5(c)). Cx37 overexpression can promote the expression of chemokine CCL7, and the expression of CCL7 decreased after the addition of Kv1.3 blocker (Figure 5(d)).

## 4. Discussion

Atherosclerosis (AS) is a chronic disease with continuous progression of large arteries, and its formation mechanism is characterized by the accumulation of vascular smooth cells, macrophages, and lipids under the intima of arteries in the early stage, followed by the formation of fibrous caps with distinct characteristics by the hyperplasia of connective tissues such collagen, elastic fibers, and proteoglycan [18]. Gap junction is a membrane channel structure between adjacent cells, and its basic constituent unit is connexin (Cx). In the early stage of atherosclerotic lesions, monocytes and vascular endothelial cells regulate each other by secreting or expressing various adhesion molecules and chemokines [19]. A large number of studies in recent years have shown that gap junction intercellular communication (GJIC) dysfunction caused by Cx expression changes is closely related to AS vascular lesions [20]. Kwak et al. [21] detected AS plaques in mice and humans and found that with the progression of AS, Cx37 expression was gradually lost in endothelial cells, gradually increased in smooth muscle, and increased in monocytes. In situ hybridization revealed abundant Cx37 expression in macrophages and foam cells in the AS region [22]. In this study, Cx37 was detected to be highly expressed in macrophages of AS mice, which was consistent with literature results.

Chemokines are secreted by endothelial cells or activated monocytes and can mediate migration and infiltration of a large number of monocytes into intima [23]. In recent years, studies have found that chemokines not only play a role in cell recruitment but also affect cell homeostasis and activation [24]. CCL7 (Chemokine C-C Motif Ligand 7) plays an important role in the recruitment of monocytes, calcium influx, tumor metastasis, and other biological effects [25]. In AS, the high expression of CCL7 can promote the recruitment and infiltration of inflammatory cells, which is directly related to the accumulation of macrophages at plaque lesions and the formation range of AS lesions and even directly related to the incidence of coronary heart disease or myocardial infarction [26, 27]. Therefore, reducing the secretion of CCL7 in monocytes can reduce the aggregation of macrophages and slow down the area of plaque formation.

In this study, we detected that Cx37 and Kv1.3 were overexpressed in macrophages of AS mice, and after Cx37 expression was silenced, the expressions of Kv1.3 and CCL7 were decreased, and the proliferation of macrophages were decreased. Wild-type mice and AS model mice were treated with Cx37 overexpression and Kv1.3 pathway blocking, and it was found that Cx37 overexpression could improve the blood lipid and blood glucose levels and increase the area of AS in AS mice. However, blocking the activity of Kv1.3 pathway can reduce the levels of blood lipid and blood glucose, increase the body weight of mice, and reduce the area of AS plaque. Blocking the activity of Kv1.3 pathway can slow down the plaque development of AS mice and make its indexes close to wild-type mice. And the use of Kv1.3 pathway blockers on the basis of overexpression of Cx37 indicated that inhibition of Kv1.3 pathway activity did not affect the expression of Cx37, but could inhibit the collagen content in the plaque area of AS mice, inhibit the expression of CCL7, and reverse the effect of Cx37 overexpression. Therefore, Cx37 can improve the activity of macrophages in AS mice by regulating the expression of chemokines and the activity of the Kv1.3 pathway and enrich macrophages in inflammatory tissues to expand the area of plaque formation.

Relevant clinical studies have shown that increasing the expression of Cx37 and the activity of chemokine CCL7 can accelerate the inflammatory reaction and promote the process of AS [10, 27], which is consistent with the conclusion of this study. To explore the inhibition of Cx37 and CCL7 expression has important significance for the treatment of atherosclerosis and provides a new therapeutic target for the treatment of atherosclerosis. At the same time, because atherosclerosis exists in a variety of diseases such as Coronary heart disease, cerebral infarction, peripheral vascular disease, targeting Cx37, Kv1.3, and other genes may have therapeutic effects on coronary heart disease and other diseases. This study proved that the expression of Cx37 can regulate the activity of Kv1.3 pathway. However, the mechanism of Cx37 regulating Kv1.3 pathway is still unclear, and further research is needed to explore the regulatory mechanism in the future.

## Figures and Tables

**Table 1:** Primer sequences of RT-PCR.

Gene	Gene ID	Primer sequence (5′-3′)		DALP (bp)
M-actb	NM_007393.5	Forward:	GAGGTATCCTGACCCTGAAGTA	104
		Reverse:	CACACGCAGCTCATTGTAGA	

M-Cx37	NM_008120.3	Forward:	CACTGGCTGCTTACCAGAAT	87
		Reverse:	CGAGGGTTCACAGAACACTTAG	

M-Kv1.3	NM_008418.2	Forward:	GTGACCATAGGAGGCAAGATT	111
		Reverse:	CCGGTGGTAGAAGTAGTTGAAG	

## Data Availability

The data used to support the findings of this study are included within the article.
